# Multiplexed DNA Methylation Analysis in Colorectal Cancer Using Liquid Biopsy and Its Diagnostic and Predictive Value

**DOI:** 10.3390/cimb43030100

**Published:** 2021-10-03

**Authors:** Walter Pulverer, Kristi Kruusmaa, Silvia Schönthaler, Jasmin Huber, Marko Bitenc, Thomas Bachleitner-Hofmann, Jagdeep Singh Bhangu, Rudolf Oehler, Gerda Egger, Andreas Weinhäusel

**Affiliations:** 1 Molecular Diagnostics, AIT Austrian Institute of Technology GmbH, 1210 Vienna, Austria; silvia.schoenthaler@ait.ac.at (S.S.); jasmin.huber@ait.ac.at (J.H.); andreas.weinhaeusel@ait.ac.at (A.W.); 2 Universal Diagnostics S.L., 41013 Seville, Spain; marko.bitenc@geneplanet.com; 3 Geneplanet d.o.o., 1000 Ljubljana, Slovenia; 4 Department of Surgery, Medical University of Vienna, 1090 Vienna, Austria; thomas.bachleitner-hofmann@meduniwien.ac.at (T.B.-H.); JagdeepSingh.Bhangu@gesundheitsverbund.at (J.S.B.); rudolf.oehler@meduniwien.ac.at (R.O.); 5 Clinical Institute of Pathology, Medical University of Vienna, 1090 Vienna, Austria; gerda.egger@meduniwien.ac.at; 6 Ludwig Boltzmann Institute Applied Diagnostics, 1090 Vienna, Austria

**Keywords:** DNA methylation, colorectal cancer, biomarker, liquid biopsy, cfDNA

## Abstract

Early diagnosis of colorectal cancer (CRC) is of high importance as prognosis depends on tumour stage at the time of diagnosis. Detection of tumour-specific DNA methylation marks in cfDNA has several advantages over other approaches and has great potential for solving diagnostic needs. We report here the identification of DNA methylation biomarkers for CRC and give insights in our methylation-sensitive restriction enzyme coupled qPCR (MSRE-qPCR) system. Targeted microarrays were used to investigate the DNA methylation status of 360 cancer-associated genes. Validation was done by qPCR-based approaches. A focus was on investigating marker performance in cfDNA from 88 patients (44 CRC, 44 controls). Finally, the workflow was scaled-up to perform 180plex analysis on 110 cfDNA samples, to identify a DNA methylation signature for advanced colonic adenomas (AA). A DNA methylation signature (*n* = 44) was deduced from microarray experiments and confirmed by quantitative methylation-specific PCR (qMSP) and by MSRE-qPCR, providing for six genes’ single areas under the curve (AUC) values of >0.85 (*WT1*, *PENK*, *SPARC*, *GDNF*, *TMEFF2*, *DCC*). A subset of the signatures can be used for patient stratification and therapy monitoring for progressed CRC with liver metastasis using cfDNA. Furthermore, we identified a 35-plex classifier for the identification of AAs with an AUC of 0.80.

## 1. Introduction

Colorectal cancer (CRC) belongs to the four big cancer entities together with breast, prostate and lung cancer, and is the third most common cancer worldwide and with around 700,000 deaths/year also one of the leading causes of cancer-mortality worldwide [1,2]. In 2017, 3857 people in Austria were newly diagnosed with CRC [3]. A look at the incidence rates of different countries reveal that CRC is three times more prevalent in developed countries than in developing countries [4], whereas the majority (70–80%) of CRC is sporadic without hereditary components [5,6]. CRC related mortality rates decreased in developed countries during recent years, which is amongst other things a direct consequence of the screening programs of the different countries [7,8,9]. However, early identification of CRC based on minimal invasive methods is still a challenge. Early identification is of utmost importance as CRC is usually asymptomatic in its early stages and therefore often diagnosed at late stages, which is associated with unfavourable outcome/prognosis [7]. As in other cancers, early diagnosis would prevent up to 90% of CRC-related deaths [2].

Molecular features of CRC include high frequency of chromosomal instability as well as hypermutation and microsatellite instability caused by impairment in DNA mismatch repair [10,11]. Like in other human neoplasms, CRC aetiology is not only driven by genetic mutations, but also by epigenetic events. Methylation of the C5’-position of cytosines in the CpG-context is the best investigated epigenetic alteration in mammalians. Its impact on embryonal and disease development is well understood and accepted [12]. Moreover, aberrant DNA methylation is recognized as a possible hit in Knudson’s two-hit theory and a hallmark of cancer, whereas specific methylation patterns for different types of cancer have been observed [13,14,15]. Epigenetic changes are already present in non-malignant and pre-malignant cells [16,17,18,19] and can be detected up to years before tumour onset. It has been well described how DNA methylation signatures are—once identified—tumour-specific and are recognized as promising targets for biomarkers for predictive as well as diagnostic purposes [20].

Many efforts have been made to develop predictive and diagnostic markers. One of the best known and best studied diagnostic methylation biomarker for CRC is SEPT9, which has been commercialized by Epigenomics. One of the pitfalls of this marker is its low sensitivity, and studies showed that its performance is better in advanced stages (III-IV) than in early stages (I-II) [21]. Combining SEPT9 test with other biomarkers (e.g., SHOX2 and ALX4) increases the sensitivity to >97%. However, these data are controversially discussed in the literature, as a meta-study found lower level of sensitivity and specificity [22]. Other diagnostic biomarkers are discussed in the literature (e.g., VIM) [23], but all of them has their limitations. According to a review of Jung et al. predictive as well as prognostic epigenetic biomarkers are under discussion in the literature, however none of them are currently relevant in clinical practice [21]. Thus, more efforts must be made in translational research to bring epigenetic biomarkers into the clinics.

Different methods are available for the readout of DNA methylation. The gold standard in that field is the bisulfite-based approach, where a bisulphite solution is applied to the DNA, which deaminates unmethylated cytosines to uracil, while methylated cytosines are protected from deamination. This approach establishes an artificial C/T mutation for all unmethylated cytosines in the genome, which can be detected by e.g., (q)PCR. In this study we exclusively use the bisulfite based approach coupled with quantitative methylation-specific PCR (qMSP).

In the presented study, we use a method based on methylation-sensitive restriction enzymes (MSRE) that cleave the DNA at distinct cutting sites, depending on the methylation status of the cytosine. Unmethylated cytosine are cleaved, while methylated ones are protected from cleaving. This results in specific amplification of methylated alleles.

In a previous study we identified 44 targets, able to discriminate CRC, blood and adjacent normal tissue, using an in-house designed DNA methylation microarray targeting 360 cancer associated genes [20,24]. Analysis of the data revealed that subsets of these 44 CRC markers have great potential for different diagnostic and predictive questions (e.g., therapy response prediction, early diagnosis of CRC). Moreover, in a recent study we investigated epigenetic alterations in rectal cancer. Rectal cancer contributes to around one-third of cases [25,26]. This study provided evidence, that specific DNA methylation signatures can be observed, depending on the location of the tumour in the colon, which is also might be of diagnostic interest. We also confirmed that our approach, based on methylation-sensitive restriction enzymes (MSREs) can generate congruent results compared to bisulfite-based approaches, which constitute the gold standard in DNA methylation analysis.

Based on our prior studies, we investigated the suitability for early diagnostics or therapy monitoring of advanced tumour stages of the 44 previously defined DNA methylation markers in combination with cfDNA extracted from liquid biopsies.

## 2. Materials and Methods

### 2.1. Patient Samples, DNA Isolation, Bisulfite Conversion and Methylation Sensitive Restriction

Patient samples were provided by the Medical University of Vienna (number of ethic votum: EK136/210 and 969/2011) and by Universal Diagnostics (2014PI/155).

Samples were taken from patients with primary colorectal cancer at the time of diagnosis as well as patients with colorectal cancer liver metastasis before and throughout systemic chemotherapy. We obtained written informed consent from the patients. The response to therapy was assessed by tumour regression grading (TRG) according to Rubbia-Brandt et al. [27] Patients with a TRG >3 were regarded as non-responders. DNA from tissue and blood samples was isolated using the Roche High Pure PCR Template Preparation Kit. cfDNA from plasma samples was isolated with Qiagen’s ccfDNA Midi Kit using 2 mL of plasma. For bisulfite conversion, the Zymo EZ DNA methylation kit was used. All kits were used according to the recommended standard protocol. Methylation sensitive digestion of the DNA was undertaken as described in [20]. In brief, 4 methylation sensitive restriction enzymes (HpaII, Hin6I, AciI, HpyCH4iV), cleaving only unmethylated DNA, were used to digest the DNA to enrich for methylated alleles. This results in a specific enrichment of methylated alleles in downstream qPCR.

### 2.2. Targeted DNA Methylation Microarray

DNA methylation analysis in 18 tumour samples and 18 adjacent tissue samples and 11 PBMC samples were performed with AIT’s in house produced targeted CpG-360 DNA methylation array. The procedure is described in [28]. In brief, 600 ng DNA were digested with a combination of 4 different methylation sensitive restriction enzymes (HpaII, Hin6I, AciI, HpyCH4IV) to cleave unmethylated DNA. Target enrichment was undertaken by multiplex PCR using assays with biotinylated reverse primers. Hybridization of the enriched target was undertaken at 52 °C over night in a hybridization oven. Detection of hybridized and therefore methylated targets was undertaken with a streptavidin-Cy3-conjugate. Readout of raw data was undertaken on an Axon 4000A microarray scanner and the GenePix 6.0 software.

### 2.3. Quantitative Methylation-Specific Polymerase Chain Reaction (qMSP)

Quantitative methylation specific PCR (qMSP) assays were designed for the targets of *TMEFF2, TWIST1, PITX2, TFPI2, DCC and PTGS2*. Primers targeting the methylated allele of the targets were designed using MethPrimer (www.urogene.org, accessed on 13. June 2018). The intercalating dye EVA Green was used for quantification. 2 µL of the bisulfite treated DNA was applied to a mastermix, consisting of 1 µL Taq Puffer 10 × including 15 mM MgCl_2_ (Qiagen, Hilden Germany); 0.8 µL dNTP mix, 2 mM each dNTP (Thermo Fisher Scientific, Massachusetts, US); 0.5 µL Eva Green 20× (Biotium, San Francisco, USA); 0.3 U HotStar TAQ-Polymerase (Qiagen, Hilden Germany). The volume per reaction was filled up with water to a total volume of 10 µL. Primer sequences are available on request. The PCR program contained a hot start for the polymerase at 95 °C for 15 min, followed by 45 cycles of 95 °C for 20 s, 55 °C for 80 s, and 72 °C for 40 s.

### 2.4. µ-Fluidic High Throughput Quantitative Polymerase Chain Reaction (qPCR) Testing of cfDNA for DNA Methylation Readout

For cfDNA methylation analysis a microfluidic high-throughput qPCR (µHT-qPCR) system was used (Fluidigm’s Biomark, San Francisco, USA) in combination with MSRE digested DNA. Assays were designed using Primer3 software, ensuring that at least two cut sites for the MSREs were in the target region. qPCR was performed with MSRE digested (75% of the eluate) and undigested DNA (25% of the eluate) for normalization purposes. Digested and undigested DNAs were subjected to a multiplexed preamplification for 22 cycles containing all 48 primerpairs (44 markers and 4 control assays). The Biomark device allows the simultaneous investigation of up to 96 samples times up to 96 target regions, which measures up to 9216 qPCR data points in a single run. For the present study, a smaller device covering 48 samples and 48 targets (=2304 reactions) was used. For the preamplification step a primer mix was prepared, consisting of the 48 primerpairs combined and diluted to a final concentration of 200 nM each primer. We combined 2.5 µL TaqMan PreAmp Master Mix (Applied Biosystems, Foster City, California), 1.25 µL of the prepared 200 nM primer mix and 1.25 µL (25 ng) of the digested DNA for the preamplification reaction. The following PCR program was used to preamplify the targets of interest: 95 °C for 10 min, 17 cycles at 95 °C for 15 s and 65 °C for 4 min. After preamplification the reactions were diluted 1:5 with H2O. Two mixes were prepared for the readout of the enriched targets. First, a sample mix, containing 2 µL of the diluted sample from the preamplification, 0.25 µL 20× Eva Green (Biotium, California, USA), 0.25 µL 20× DNA binding dye sample loading reagent (Fluidigm, San Francisco, USA), and 2.5 µL 2 × TaqMan Gene Expression Master Mix (Applied Biosystems, Foster City, California) was prepared. Second, an assay mix containing 2.5 µL assay loading reagent (Fluidigm, San Francisco, USA), 0.25 µL H2O and 2.25 µL of pooled forward and reverse primer (20 µM each primer) was prepared. The two mixes were applied to Fluidigm’s 48.48 GE Dynamic Array according to the manufacturer’s protocol. Cycling conditions were as follows: thermal mixing: 50 °C for 2 min, 70 °C for 30 min, 25 °C for 10 min; hot start: 95 °C 15 min; followed by 35 cycles at 95 °C for 40 s, 65 °C for 40 s and 72 °C for 80 s; final elongation: 72 °C 7 min. Finally, a melting curve measurement was undertaken.

### 2.5. Bioinformatics and Statistics

Statistical evaluation of the array was performed and qPCR data taken using BRB Array tools Version 4.6.1 (https://brb.nci.nih.gov/BRB-ArrayTools/, accessed on 16 November 2018) and RStudio Version 1.3.1093 (https://www.rstudio.com/, accessed on 20 October 2020) with R statistical software Version 4.0.3 (https://www.r-project.org/, accessed on 20 October 2020). Data derived from microarray and qPCR experiments were quantile normalised and methylation differences between the different groups were investigated by random-variance t-test and comparative analysis.

Statistical workflow for AA study: (1) on training set, features were ranked according to their variance of importance (VIP) using a random forest feature selection algorithm with Monte-Carlo cross-validation over 50 sub-setting iterations, with 2/3 of the data used for training of the model and 1/3 of the data for testing of the model; (2) VIP values over 50-iterations were averaged, creating a consensus list; (3) markers with VIP >2 were further used for support-vector machine (SVM) algorithm building; (4) SVM model was applied on Testing Set; (5) pROC package was used for establishing sensitivity, specificity and area under the curve (AUC) values.

## 3. Results

### 3.1. Identification of Differentially Methylated Targets between Colorectal Cancer (CRC), Adjacent Tissue and Blood from Healthy Individuals

**Figure 1:**
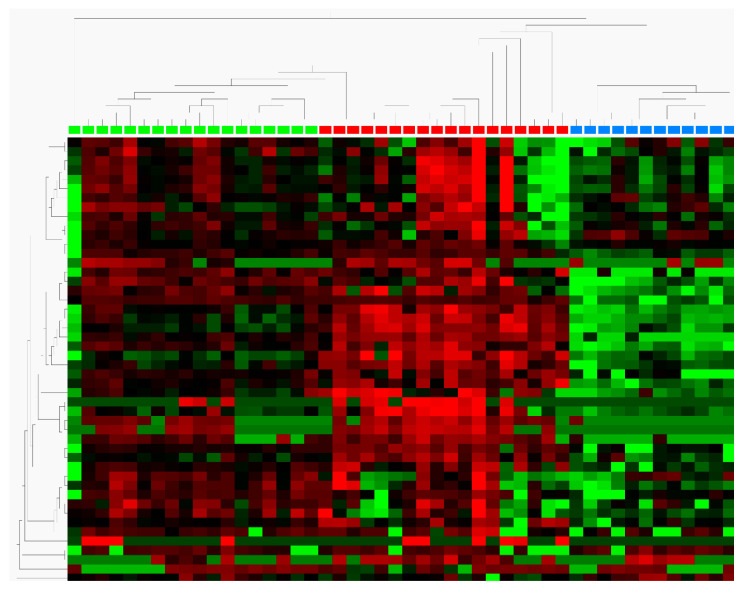
Heatmap showing the distinct methylation profiles of adjacent tissue (green), colorectal cancer (CRC, red), and PBMCs (blue). A clear separation between the sample indicates the presence of a specific methylation signature for CRC.

In previous experiments we defined a 44-plex DNA methylation signature in colorectal tumours using an in house produced DNA methylation array targeting regions of 360 cancer-associated genes [29]. Technical validation of those 44 targets in 18 fresh frozen colorectal tumours, 18 fresh frozen adjacent tissues and 12 blood samples from healthy volunteers was performed by high-throughput MSRE qPCR. A two-sided *t*-test was performed to check for differential methylation. We identified 25 targets with differential methylation in the group comparison of tumour vs. PBMC samples. Of those, 11 were highly significant (*p* < 10^−4^) with log2-fold-changes between 1 and 19. For the group comparison tumour vs. adjacent normal tissue, we identified 19 differential methylated targets with *p* < 10^−4^ and a log2-fold-change between 2 and 5. Seventeen of the targets showed differential methylation behaviour in both comparisons (Table 1). As expected, we identified more differentially methylated targets in the contrast tumour vs. blood (*n* = 25; *ESR1*, *TFPI2*, *WT1*, *TMEFF2*, *PENK*, *MYOD1*, *TWIST1*, *DCC*, *PTGS2*, *TJP2*, *SPARC*, *PITX2, SEZ6L*, *DNAJC15*, *GDNF*, *CDX1*, *CLIC4*, *SFRP2*, *HLA-G*, *GATA4*, *BOLL*, *THBD*, *RARB*, *NKX2-1*, *SALL3*) compared to the contrast tumour vs. adjacent tissue (*n* = 19, *ESR1*, *TFPI2*, *WT1*, *TMEFF2*, *PENK*, *MYOD1*, *TWIST1*, *DCC*, *PTGS2*, *TJP2, SPARC*, *PITX2*, *SEZ6L*, *DNAJC15*, *GDNF*, *CDX1*, *CLIC4*, *SFRP2*, *HLA-G*, *GATA4*, *BOLL*, *THBD*, *RARB*, *NKX2-1*, *SALL3*, *TCEB2*, *S100A8*). The heatmap (Figure 1) shows a graphical summary for all the included 44 genes and provides evidence for the distinct methylation signature for CRC. Hierarchical clustering resulted in partitioning of the data into three clusters, separating CRC samples from PBMC and normal adjacent tissue samples. All 25 targets were found to be hypermethylated in CRC, with S100A8 as the only exception, which showed hypomethylation in CRC.

### 3.2. Validation of the Methylation-Sensitive Restriction Enzyme (MSRE)-Based Approach by MSP as a Gold Standard

The second set of questions aimed to compare the data generated with MSRE based methods with data generated with bisulfite-based methods, which are recognized as gold standard in the field. For that purpose we selected four targets (*TMEFF2* (*p* < 10^−7^), *PITX2* (*p* < 10^−7^), *TWIST1* (*p* < 10^−7^), *TFPI2* (*p* < 10 × 2.3^−6^)) with highly significant differential methylation; *DCC* with moderate significant differential methylation (*p*= 0.014) and *PTGS2* lacking differential methylation (*p* = 0.29) between the tumour group and the adjacent tissue group. DNA isolated from fresh frozen tissue (CRC *n* = 71; control *n* = 65) was used for the MSRE-based qPCR and DNA isolated from FFPE material (CRC *n* = 58; control *n* = 48) was used for the bisulfite-based qPCR. The use of FFPE DNA for bisulfite-based qPCR was a compromise, as the available DNA from fresh frozen tissue was exhausted.

**Figure 2:**
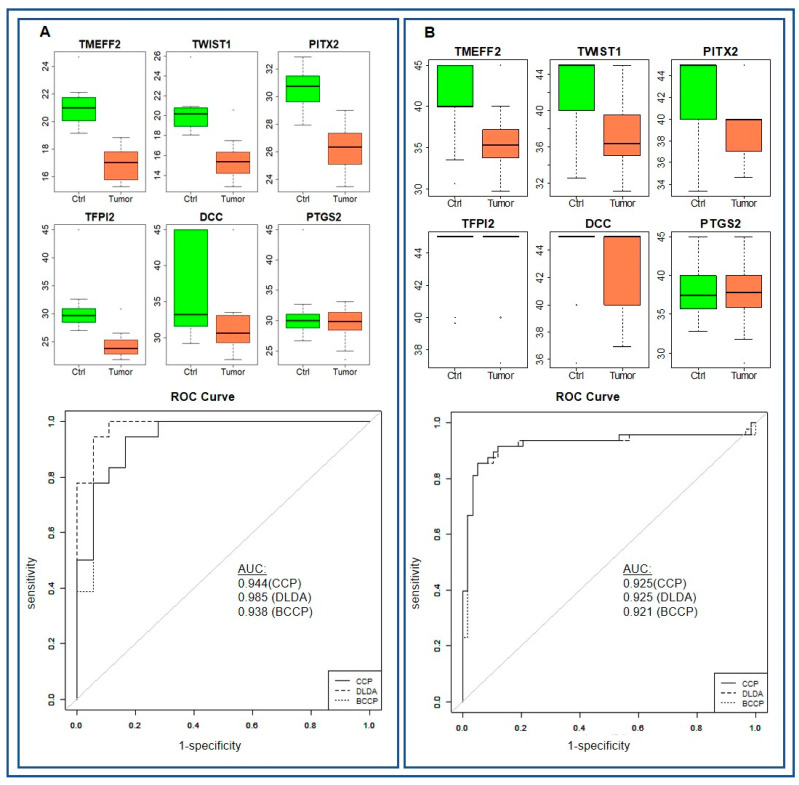
Boxplot and receiver operating characteristics (ROC) curves for the comparison of MSRE (**A**) and qMSP (**B**) generated data.

*TMEFF2*, *PITX2* and *TWIST1* provided with both methods identical *p*-values (*p* < 10^−7^). For TFPI2 neither MSRE nor MSP could provide cp-values, as can be seen in Figure 2B boxplot. Only differential methylation for *DCC* (*p* = 0.2366) was not confirmed by MSP. No differential methylation was found for *PTGS2* with the bisulfite based method, confirming the MSRE data. The differences in the fold-change between the groups of up to 36.6-fold can be explained by the use of FFPE material, which is known to impair DNA quality (Table 2 and Figure 2). Consequently, we were able to reproduce the data with the gold standard method for 5 of 6 markers. In summary, these results show on the one hand that we have selected a panel of 48 different targets having a specific DNA methylation pattern in CRC, and on the other hand that the MSREqPCR based method is an alternative to bisulfite-based methods. Additionally, MSREqPCR allows for high-throughput multiplexed reactions (up to 96plex) using µ-fluidic qPCR, which enables the parallel analysis of a multitude of samples and markers, as discussed below.

### 3.3. Potential of the 44plex Methylation Panel for Early Identification of CRC

**Figure 3:**
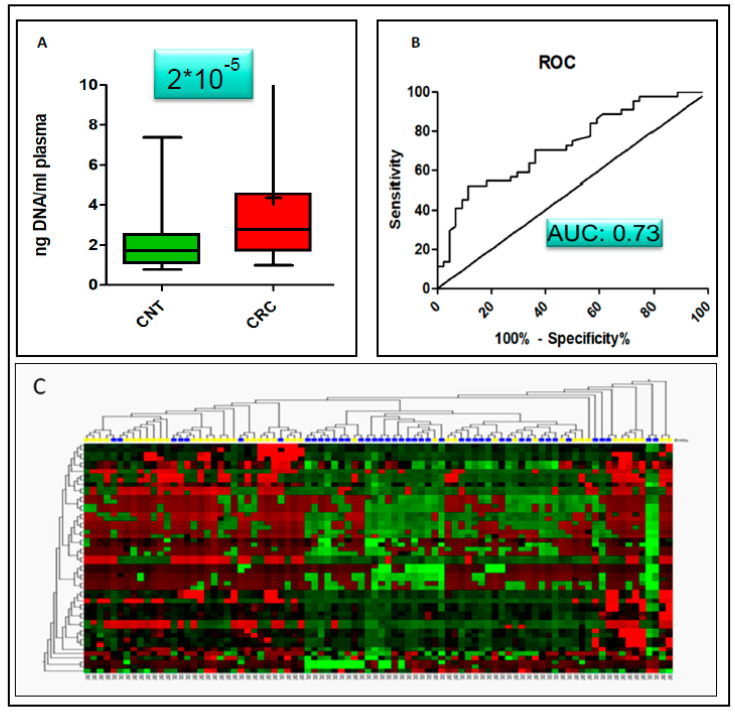
Diagnostic value of cfDNA yield. Statistical comparison of the isolated amount of cfDNA from CRC and control non-tumour samples (CNT) provided evidence that the amount of isolated cfDNA is a strong indicator for the presence of a tumour. (**A**): Increased amounts of cfDNA (4.36 +/− 4.82 ng DNA/mL plasma) were found in CRC compared to CNT (2.06 +/− 1.27 ng DNA/mL plasma), which are statistically significant (*p* = 2 × 10−5). (**B**): single AUC value was 0.73. (**C**): Heatmap with hierarchical clustering for CRC and CNT samples. Two major clusters with prevalence for either CRC or CNT are present. However, 8 CNT and 5 CRC samples did not cluster to the correct group, maybe indicating samples with an intermediate state.

The next section of the study was concerned with the evaluation of the suitability of the selected 48plex panel for early CRC diagnostics using liquid biopsies. We followed a strategy, which allowed for the isolation of cfDNA using at least 2ml plasma per sample combined with small (20 µL) elution volumes. One of the interesting aspects is that samples collected from early CRC patients have an elevated concentration of cfDNA (*n* = 44; ng DNA/mL plasma: 4.36 + /−4.82) yield compared to the control group (*n* = 44; ng DNA/mL plasma: 2.06+/−1.27) and could therefore be used as an additional indicator for onset of CRC. This result is significant at the *p* = 0.0002 level Figure 3A and an area und the curve of 0.73 in receiver operating characteristics (ROC; Figure 3B).

Biostatistical evaluation using delta cp (dcp) normalized data (dcp = cp_digested − _cp_undigested_) revealed that differential methylation in early CRC samples is present in 20 of the 48 investigated targets (*PITX2, DCC*, *TMEFF2*, *TWIST1*, *MYOD1*, *SPARC*, *TP53*, *WT1*, *CXADR*, *SERPINB2*, *S100A2*, *SRGN*, *PITX2*, *PENK*, *CDX1*, *BOLL*, *NKX2-1*, *TFPI2*, *DAPK1*, *THBD)* at a significance level of *p* < 0.05 (Table 3). All (20 out of 48) reported targets in Table 3 were found to be hypermethylated in CRC with a fold-change between 1.04 and 5.43. The methylation signature allows for a correct classification of 78% of the samples using the diagonal linear discriminant prediction (DLD) algorithm with an area under the curve of 0.85 According to DLD 33 out of 44 CRC samples and 36 out of 44 controls (CNT) were correctly classified using Leave-on-out-cross-validation. This result implies a specificity of 0.75 and a sensitivity of 0.82. The positive predictive value was 0.77, while the negative predictive value was 0.80. Individual AUC calculations showed that 6 genes had values of AUC >0.8 (DAPK1: 0.875; WT1: 0.85; PENK: 0.85: DCC 0.83; PITX2: 0.82; TMEFF2: 0.82; Table 4). Hierarchical clustering analysis for CRC and CNT was undertaken using MSREqPCR dcp values of the 44 targets and showed 2 major distinct clusters with prevalence for either CNT (26 out of 44) or CRC (32 out of 44) samples. Eight CNT and 5 CRC samples that did not cluster with one of the two major groups (Figure 3C), might represent samples with an intermediate state between CNT and CRC.

### 3.4. cfDNA Methylation Analysis of Progressed CRC with Liver Metastasis for Therapy Response Prediction

**Figure 4:**
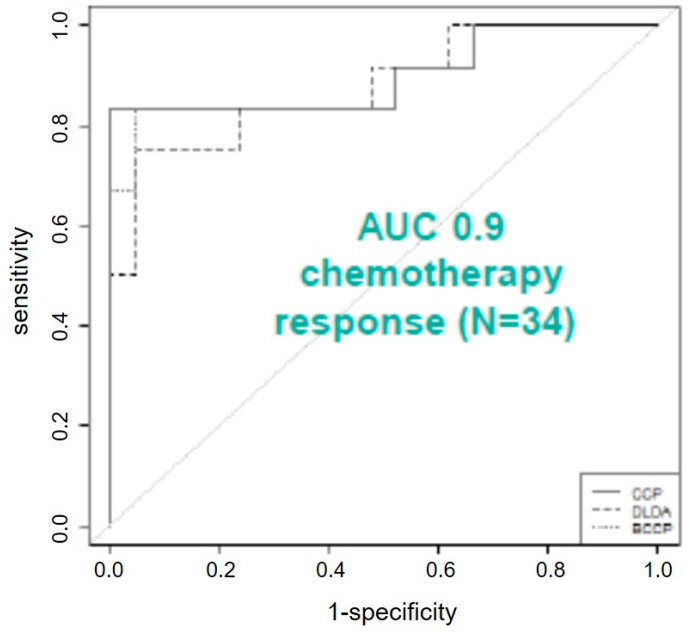
Receiver operating characteristics provided an AUC of 0.9 for chemotherapy response prediction in patients with CRC liver metastasis.

Following our successful confirmation of a set of DNA methylation markers for early detection of CRC in liquid biopsies, we set out to determine the suitability of our marker panel for therapy response prediction to neoadjuvant chemotherapy in CRC patients with liver metastases. For this, we measured DNA methylation from 33 different patients (responder *n* = 12; non-responder *n* = 21) over three different time points. Notably, we found 26 of the 44 targets with differential methylation between responders and non-responders in one or more of the three measured time points (number of differentially methylated genes in time point 1: *n* = 13; number of differentially methylated genes in time point 2: *n* = 17; number of differentially methylated genes in time point 3: *n* = 9). *CDX1*, *PITX2* and *TBP* showed differential methylation over all three time points, *CD24*, *SERPINB2*, *WT1 FMR1*, *IL1B*, *S100A2*, and *TP53* were differentially methylated in two time points. Differential methylation in one time point was observed for the genes *BOLL*, *ESR1*, *HLA-G*, *MYOD1*, *PTGS2, TMEFF2*, *TWIST1*, *CALCA*, *H19*, *MSH4*, *RARB*, *SFRP2*, *SPARC, TCEB2*, *TFPI2* and *THBD* (Table 5). Further analysis, using quantitative trait analysis (based on a Spearman correlation) correlating our methylation markers with data from the commercially available SEPT9-kit (Epigenomics, Germany) showed that 16 of the investigated targets had a correlation >0.6 with SEPT9 and six genes had a correlation of >0.9 (*MYOID1*, *WT1*, *SFRP2*, *SALL3*, *GDNF* and *ESR1*; Table 6). The most striking result to emerge from the data is that a combination of three targets (*BOLL*, *DCC*, *SFRP2*) can discriminate between responders and non-responders during adjuvant chemotherapy is administered and subsequently support the decision regarding the need for an operation. These three targets provide a classifications success in ROC-curve analysis of AUC 0.9 (Figure 4) and have impressive high single AUC values (*BOLL* AUC = 0.87; *DCC* AUC = 0.92; *SFRP2* AUC = 0.91) as well as good correlation with the methylation data of *SEPT9* (*BOLL* R = 0.89; *DCC* R = 0.7; *SFRP2* = 0.93). Those AUC-values outperform the received AUC-value of the commercially used marker *SEPT9*, which showed in our experiments an AUC value of 0.86, still being remarkable. Three nearest-neighbors analysis showed an alignment success of the samples to the correct group (operated; non-operated) of 88% using the three targets *BOLL*, *DCC* and *SFRP2*. This resulted in sensitivities of 0.95 and sensitivities of 0.75. Positive prediction value was 0.87, while the negative prediction value was 0.9. This indicates that we can identify with high accuracy those patients who should undergo surgery with high accuracy.

### 3.5. Early Cancer Diagnostics of Advanced Adenomas (UDX, 180 Marker Times 110 Samples)

Based on the high sensitivity and specificity of DNA methylation markers in early CRC diagnostics, we expanded our analyses to DNA methylation analysis of liquid biopsies for early diagnostics of advanced colonic adenomas (AA). For this purpose, we selected a new set of 180 target regions, which were derived from whole genome bisulfite sequencing (WGBS) analysis of three pooled plasma samples consisting of colonoscopy verified control samples, three AA samples and four consisting of CRC samples. WGBS analysis resulted in the selection of putative biomarker regions beyond previously described promoter regions, covering also gene desert regions.

We evaluated 180 candidate regions in individual pre-colonoscopy plasma samples prospectively collected from 110 patients. Performance of the methylation marker panel was tested by dividing the sample set into a training set (24 AA patients and 30 age, gender and BMI matched control patients) and a testing set (16 AA and 40 age, gender and BMI matched control patients) using machine learning algorithms.

**Figure 5:**
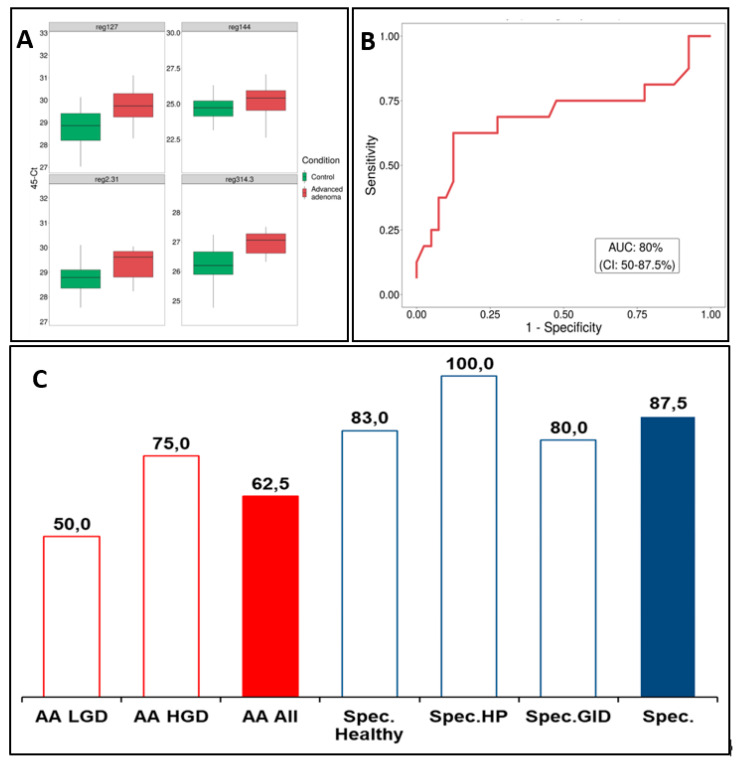
(**A**) Detection of methylation markers in plasma. (**B**) ROC curve and AUC for 35-marker panel performance on the Testing set that includes samples of advanced adenoma, healthy, HP (patients with histologically confirmed hyperplastic polyp findings) and GID (patients found during colonoscopy to have ulcerative colitis or IBD) patients. (**C**) 35-marker panel performance. Accuracy values for 35-marker prediction on the Testing set, where red bar represents the overall sensitivity for advanced adenomas (AA AII), red outlined bars represent sensitivity for advanced adenoma with low grade dysplasia (AA LGD) and advanced adenoma with high grade dysplasia (AA HGD) and blue bar represents specificity and blue outlined bars represents specificity separately for GID group, high group and healthy group.

A support vector machine (SVM) model, built on the training set utilized the 35 most discriminate methylation markers (details for the genomic regions can be found in Supplemental Table S1). SVM model performance on the testing set achieved an AUC of 0.80 (Figure 5B). The sensitivity for detecting any AA was 0.63, meaning that 10 of the 16 adenomas of the testing set were correctly identified at a specificity of 0.88. AA sub-class analysis showed that detection of most severe type of AA, high-grade dysplasia patients, had superior sensitivity of 0.75 (6/8 correctly identified) compared to 0.50 for low grade dysplasia detection (4/8). Sensitivity for tubulovillous adenoma patients was 0.63 (6/8) and for tubular adenoma 0.50 (3/6), while singular serrated and villous adenoma cases were both correctly classified. Furthermore, 100% of patients with hyperplastic polyps and 83% (15/18) of colonoscopy negative control patients were correctly identified as controls (Figure 5C). The four top markers depicted in Figure 5A indicate hypermethylation in the AA patients as compared to control patients. These results indicate that cfDNA methylation panels could be used for the identification of patients with early pre-cancerous adenomas with good accuracy.

## 4. Discussion

Several reports have shown that management of CRC is still a challenge due to its asymptomatic course in the first phases of the disease, making it an undetected disease at a time where clinical intervention would be most efficient. Therefore, a focus in management of CRC should be on the early detection of the disease, ideally before tumour onset, in AA state. In line with that, epigenetic changes, which are important and early events in tumorigenesis, have great potential for CRC-disease management. Our previous studies on DNA methylation in CRC tissue identified specific epigenomic alterations, deducing a DNA methylation signature able to discriminate neoplastic tissue from adjacent tissue and from blood [20,24,29]. An initial objective during our CRC-related research was to refine and further develop this DNA methylation signature, aiming to provide methods based on liquid biopsies for early and minimal invasive tumour detection as well as monitoring purposes. An important question in this setting sought to validate the MSRE-based DNA methylation readout method with a bisulfite based approach. As a result, this study found that MSRE based DNA methylation testing correlates well with bisulfite based DNA methylation testing. One unanticipated finding was that MSRE-based testing provided more fine-tuned view on the methylation differences of the tested groups (control vs. cancer). While bisulfite-based testing detected methylation levels around 0%, the MSRE-based testing also provided an insight into the methylation levels of the control group, which is of interest when background DNA methylation levels must be determined and considered. There are two likely causes for the differences that can be discussed. Firstly, exposing DNA to bisulfite treatment is a very harsh condition, impairing overall DNA quality. This is paired with a reduction of the sequence complexity by the fraction of unmethylated cytosines. Secondly, the impaired DNA quality deriving from FFPE material is caused by formalin-induced DNA fragmentation and insufficient de-crosslinking of protein-DNA complexes during DNA isolation. This point might explain the late cp values of the bisulfite based testing compared to the MSRE-based testing.

A bisulfite-free method based on enzymes for deamination was recently presented by New England Biolabs, which is might be a promising tool and alternative to the bisulfite-based approaches for DNA methylation readout, as it is known to avoid fragmentation of the DNA and applies in general less severe conditions to the DNA. Our lab is currently testing this bisulfite-free method as alternative.

The core of this study was a detailed investigation of the identified CRC biomarkers for early diagnostics and its suitability for liquid biopsy testing. As expected, the concentration of cfDNA per ml plasma was a rather strong indicator for the presence of CRC and might allow to draw first conclusions. Notably, distinct methylation signatures consisting of a subpanel of 20 targets were already present in patients with early CRC. This observation may support the hypothesis that liquid biopsies in CRC can be used for disease monitoring/disease detection at very early stages, where surgical/clinical intervention is most efficient and can lead to complete remission of the cancer.

We also found evidence that a three-gene signature consisting of *BOLL*, *DCC*, *SFRP2* can be used for therapy stratification in CRC patients with liver metastasis to monitor the response to neoadjuvant chemotherapy. Additionally, for the CRC liver metastasis setting we found that DNA methylation markers investigated in cfDNA from liquid biopsies provided superior AUC values as the already established tumour marker *Septin9*, providing a rationale for implementing those markers in the clinical routine. Following extensive validation of our markers and our used methods, we find that the defined methylation markers are highly sensitive and specific for CRC detection in early stages of CRC and allow for the identification of responders and non/responders to neoadjuvant chemotherapy in metastatic patients. Moreover, there might be a benefit for existing commercially available biomarkers like the Septin9 in the Epi proColon Kit (https://www.epigenomics.com/products/epi-procolon/, accessed on 15 April 2021) from Epigenomics AG to improve their diagnostic relevance [30].

Another important conclusion we can draw from the presented work is that our workflow was very flexible in terms of the investigated content and allowed a fast and relatively simple change of the targets to be investigated. This was highlighted by the identification of biomarkers for the detection of advanced adenomas. In this setting, we used a new set of 180 targets, which we designed from scratch. Targets were selected based on a WGBS approach on plasma samples. The resulting 35-marker model yielded an overall sensitivity 0.63 and a specificity of 0.88, indicating the feasibility of early identification of AAs through cfDNA methylation analysis. These findings are of special interest in the context of further research into cancer prevention and monitoring or prediction of the transition from adenoma to carcinoma.

Taken together, our work strongly emphasizes the use of DNA methylation testing in liquid biopsies for future management of advanced CRC including patients suffering from metastasis, but also for patients in early or even premalignant stages. However, it is important to bear in mind that DNA methylation testing of cfDNA is still a challenging procedure, needing very experienced users and special lab equipment (e.g., Fluidigms Biomark HD) and protocols, which might not be available in routine laboratories.

Consequently, more research needs to be undertaken and standard assays need to be developed in order to overcome limitations such as the minute amounts of cfDNA in plasma or other technical restrictions.

## Figures and Tables

**Table 1:** Overview of the technically validated targets, deduced from the targeted DNA methylation microarray experiments. Technical validation of the targets was undertaken with methylation-sensitive restriction enzyme high-throughput quantitative polymerase chain reaction (MSRE-HT-qPCR) and confirmed 25 genes for the comparison tumour vs. PBMC and 19 genes for the comparison tumour vs. adjacent tissue. Differential methylation for both comparisons was found for 17 targets. PBMC = peripheral blood mononuclear cell.

Gene Symbol	Descirption	Tumour vs. PBMC			Tumour vs. Adjacent Tissue		
		*p*-Value	FDR	log2-Fold-Change	*p*-Value	FDR	Log2-Fold-Change
*ESR1*	Estrogen receptor 1	<1 × 10^−7^	<1 × 10^−7^	7	<1 x10^−7^	<1 × 10^−7^	4
*TFPI2*	Tissue factor pathway inhibitor 2	<1 × 10^−7^	<1 × 10^−7^	19	2.3 × 10^−6^	0.0000184	6
*WT1*	Wilms tumor 1	<1 × 10^−7^	<1 × 10^−7^	6	3.9 × 10^−6^	0.0000267	2
*TMEFF2*	Transmembrane protein with EGF-like and two follistatin-like domains 2	<1 × 10^−7^	<1 × 10^−7^	7	<1 × 10^−7^	<1 × 10^−7^	4
*PENK*	Proenkephalin	<1 × 10^−7^	<1 × 10^−7^	8	0.002722	0.0131	2
*MYOD1*	Myogenic differentiation 1	<1 × 10^−7^	<1 × 10^−7^	4			
*TWIST1*	Twist homolog 1 (Drosophila)	<1 × 10^−7^	<1 × 10^−7^	5	<1 × 10^−7^	<1 × 10^−7^	5
*DCC*	Deleted in colorectal carcinoma	<1 × 10^−7^	<1 × 10^−7^	12	0.014024	0.0449	4
*PTGS2*	Prostaglandin-endoperoxide synthase 2 (prostaglandin G/H synthase and cyclooxygenase)	<1 × 10^−7^	<1 × 10^−7^	12			
*TJP2*	Tight junction protein 2 (zona occludens 2)	<1 × 10^−7^	<1 × 10^−7^	15			
*SPARC*	Secreted protein, acidic, cysteine-rich (osteonectin)	4.00 × 10^−7^	1.60 × 10^−6^	5			
*PITX2*	Paired-like homeodomain 2	4.00 × 10^−7^	1.60 × 10^−6^	8	<1 × 10^−7^	<1 × 10^−7^	4
*SEZ6L*	Seizure related 6 homolog (mouse)-like	1.20 × 10^−6^	4.43 × 10^−6^	14	3.23 × 10^−3^	1.41 × 10^−2^	3
*DNAJC15*	DnaJ (Hsp40) homolog, subfamily C, member 15	1.40 × 10^−6^	4.50 × 10^−6^	11	1.71 × 10-^2^	5.11 × 10^−2^	5
*GDNF*	Glial cell derived neurotrophic factor	1.50 × 10^−6^	4.50 × 10^−6^	8	6.60 × 10^−4^	3.96 × 10^−3^	2
*CDX1*	Caudal type homeobox 1	1.50 × 10^−6^	4.50 × 10^−6^	3			
*CLIC4*		3.40 × 10^−6^	9.60 × 10^−6^	11	2.25 × 10^−2^	6.00 × 10^−2^	5
*SFRP2*	Secreted frizzled-related protein 2	3.05 × 10^−5^	8.13 × 10^−5^	12	6.31 × 10^−3^	2.52 × 10^−2^	4
*HLA-G*	Major histocompatibility complex, class I, G	3.77 × 10^−5^	9.52 × 10^−5^	1	1.56 × 10^−3^	8.32 × 10^−3^	1
*GATA4*	GATA binding protein 4	7.86 × 10^−5^	1.89 × 10^−4^	3	1.81 × 10^−2^	5.11 × 10^−2^	1
*BOLL*	Bol, boule-like (Drosophila)	9.80 × 10^−5^	2.24 × 10^−4^	7	2.00 × 10^−7^	1.92 × 10^−6^	4
*THBD*	Thrombomodulin	1.65 × 10^−4^	3.60 × 10^−4^	8			
*RARB*	Retinoic acid receptor, beta	9.28 × 10^−4^	1.94 × 10^−3^	7	9.12 × 10^−3^	3.13 × 10^−2^	5
*NKX2-1*	NK2 homeobox 1	6.80 × 10^−3^	1.36 × 10^−2^	3			
*SALL3*	Sal-like 3 (Drosophila)	2.63 × 10^−2^	5.05 × 10^−2^	4			
*TCEB2*	Transcription elongation factor B (SIII), polypeptide 2 (18 kDa, elongin B)				8.63 × 10^−3^	3.13 × 10^−2^	149
*S100A8*	S100 calcium binding protein A8				0.040431	0.102	1

**Table 2:** Comparison of the MSRE-based approach and the qMSP approach. 6 targets from the targeted DNA methylation array have been selected for validation by qMSP. TMEFF2, PITX2, TWIST1 showed high differential methylation (*p* < 1 × 10^−7^); TFPI2 and DCC showed intermediate methylation difference and PTGS showed no differential methylation. qMSP confirms the findings for 5 of the 6 targets. The intermediate differential methylation for DCC from MSRE based approach was not confirmed by qMSP.

Gene Symbol	*p*-Value (Fresh Frozen Tissue)	Fold-Change (Fresh Frozen Tissue)	*p*-Value (FFPE)	Fold-Change (FFPE)
*TMEFF2*	<1 × 10^−7^	16.95	<1 × 10^−7^	68.39
*PITX2*	<1 x10^−7^	17.86	<1 × 10^−7^	15.14
*TWIST1*	<1 x10^−7^	24.39	<1 × 10^−7^	43.28
*TFPI2*	2.3 x10^−6^	66.67	0.0127	1.82
*DCC*	0.0140	20.00	0.2366	1.47
*PTGS2*	0.2905	2.33	0.8630	1.09

**Table 3:** Differential DNA methylation was detected in 20 of the 44 targets in early CRC patients, which enables early identification of CRC. All the targets were found to be hypermethylated in CRC.

Gene Symbol	*p*-Value	Mean of	Mean of	Fold Change	Entrez ID
		dcp in Control no Tumor (CNT)	dcp in CRC		
*PITX2*	1.00 × 10^−7^	8.03	7.3	1.66	paired-like homeodomain 2
*DCC*	2.00 × 10^−7^	7.89	7.26	1.55	DCC netrin 1 receptor
*TMEFF2*	4.00 × 10^−7^	6.23	5.7	1.44	transmembrane protein with EGF-like and two follistatin-like domains 2
*TWIST1*	1.17 × 10^−5^	5.59	5.21	1.30	twist family bHLH transcription factor 1
*MYOD1*	4.68 × 10^−5^	6.53	6.08	1.37	myogenic differentiation 1
*SPARC*	3.35 × 10^−4^	6.24	5.75	1.40	secreted protein, acidic, cysteine-rich (osteonectin)
*TP53*	6.01 × 10^−4^	6.46	6.08	1.30	tumor protein p53
*WT1*	1.07 × 10^−3^	6.62	5.47	2.22	Wilms tumor 1
*CXADR*	1.23 × 10^−3^	4.71	4.53	1.13	coxsackie virus and adenovirus receptor
*SERPINB2*	1.27 × 10^−3^	5.32	5.4	0.95	serpin peptidase inhibitor, clade B (ovalbumin), member 2
*S100A2*	1.68 × 10^−3^	5.9	5.65	1.19	S100 calcium binding protein A2
*SRGN*	4.82 × 10^−3^	3.85	3.79	1.04	serglycin
*PITX2*	5.97 × 10^−3^	6.38	5.93	1.37	paired-like homeodomain 2
*PENK*	8.63 × 10^−3^	8.41	7.33	2.11	proenkephalin
*CDX1*	1.41 × 10^−2^	5.85	5.67	1.13	caudal type homeobox 1
*BOLL*	2.42 × 10^−2^	6.41	6.06	1.27	boule-like RNA-binding protein
*NKX2-1*	2.97 × 10^−2^	6.7	6.3	1.32	NK2 homeobox 1
*TFPI2*	3.13 × 10^−2^	7.75	7.24	1.42	tissue factor pathway inhibitor 2
*DAPK1*	3.32 × 10^−2^	9.39	6.95	5.43	death-associated protein kinase 1
*THBD*	3.81 × 10^−2^	7.88	6.5	2.60	thrombomodulin

**Table 4:** Single area under the curve (AUC) values for the 20 genes, showing differential DNA methylation in CRC samples.

Gene	AUC
*DAPK1*	0.8750
*WT1*	0.8508
*PENK*	0.8469
*DCC*	0.8258
*PITX2*	0.8224
*TMEFF2*	0.8196
*SPARC*	0.7921
*TWIST1*	0.7823
*MYOD1*	0.7809
*TP53*	0.7471
*S100A2*	0.7197
*TFPI2*	0.7093
*CXADR*	0.6839
*SERPINB2*	0.6771
*BOLL*	0.6694
*SRGN*	0.6614
*PITX2*	0.6539
*NKX2-1*	0.6444
*CDX1_WH*	0.6374
*THBD*	0.5865

**Table 5:** Overview of the 26 targets found with differential methylation of patients with CRC liver metastasis, responding/non-responding to neoadjuvant chemotherapy.

	Time Point 1		Time Point 2		Time Point 3	
	*p*-Value	Fold-Change	*p*-Value	Fold-Change	*p*-Value	Fold-Change
*TWIST1*	4.26 × 10^−3^	2.78				
*CDX1*	6.12 × 10^−3^	12.7	1.84 × 10^−3^	18.3	2.77 × 10^−2^	6.63
*PITX2*	7.40 × 10^−3^	6.67	1.91 × 10^−2^	13.4	1.64 × 10^−2^	2.5
*ESR1*	1.55 × 10^−2^	3.23				
*CD24*	1.59 × 10^−2^	<1 × 10^−7^			4.86 × 10^−2^	547
*BOLL*	1.61 × 10^−2^	4				
*PTGS2*	1.62 × 10^−2^	313				
*MYOD1*	2.15 × 10^−2^	2.44				
*TBP*	2.67 × 10^−2^	11.6	3.40 × 10^−4^	27.9	1.05 × 10^−3^	24.3
*WT1*	3.30 × 10^−2^	3.85	4.47 × 10^−2^	2.38		
*TMEFF2*	3.94 × 10^−2^	2.17				
*SERPINB2*	3.96 × 10^−2^	125			2.50 × 10^−2^	667
*HLA-G*	4.36 × 10^−2^	1.92				
*SFRP2*			1.81 × 10^−4^	333		
*S1000A2*			3.51 × 10^−4^	15.6	3.69 × 10^−3^	7.47
*TP53*			2.34 × 10^−3^	21.3	4.87 × 10^−3^	26.8
*THBD*			4.77 × 10^−3^	263		
*FMR1*			5.70 × 10^−3^	27,000	1.08 × 10^−3^	168,000
*TCEB2*			1.39 × 10^−2^	32.6		
*TFPI2*			2.17 × 10^−2^	9.09		
*MSH4*			2.68 × 10^−2^	2		
*CALCA*			2.68 × 10^−^	2.33		
*H19*			2.77 × 10^−2^	1.6		
*SPARC*			4.28 × 10^−2^	10		
*IL1B*			4.47 × 10^−2^	1.52	8.81 × 10^−3^	2.27
*RARB*			4.72 × 10^−2^	159		

**Table 6:** Correlation analysis with Epigenomics’ commercially available Septin-9 marker for CRC diagnostics 19 targets showed correlation with the commercially available marker SEPTIN-9 of >0.605.

Gene Symbol	Correlation Coefficient	*p*-Value
*ESR1*	0.935	<1 × 10^−7^
*GDNF*	0.906	<1 × 10^−7^
*SALL3*	0.903	<1 × 10^−7^
*SFRP2*	0.932	<1 × 10^−7^
*WT1*	0.918	<1 × 10^−7^
*MYOD1*	0.901	<1 × 10^−7^
*BOLL*	0.888	<1 × 10^−7^
*TMEFF2*	0.887	<1 × 10^−7^
*TFPI2*	0.875	1.00 × 10^−7^
*SPARC*	0.828	4.00 × 10^−7^
*TWIST1*	0.825	4.00 × 10^−7^
*SEZ6L*	0.782	8.00 × 10^−7^
*DCC*	0.7	1.03 × 10^−5^
*ZNF526*	0.696	1.25 × 10^−5^
*GATA4*	0.665	3.78 × 10^−5^
*THBD*	0.605	2.58 × 10^−4^

## Data Availability

Data is available upon request.

## Supplementary Materials

Supplementary materials can be found at https://www.mdpi.com/article/10.3390/cimb43030100/s1.
